# Intimate-Partner and Client-Initiated Violence among Female Street-Based Sex Workers in China: Does a Support Network Help?

**DOI:** 10.1371/journal.pone.0139161

**Published:** 2015-09-28

**Authors:** Katie Hail-Jares, Ruth C. F. Chang, Sugy Choi, Huang Zheng, Na He, Z. Jennifer Huang

**Affiliations:** 1 Department of International Health, School of Nursing and Health Studies, Georgetown University, Washington, D.C., United States of America; 2 Department of Epidemiology, Mailman School of Public Health, Columbia University, New York, New York, United States of America; 3 Shanghai Piao Xue, Shanghai, China; 4 Department of Epidemiology, School of Public Health, Fudan University, Shanghai, China; David Geffen School of Medicine at UCLA, UNITED STATES

## Abstract

**Background:**

Globally, female street-based sex workers are vulnerable to gender-based violence. Previous research has shown having a peer social network can reduce sex workers’ risks of victimization. However, mechanisms of how social network impacts violence among female street-based sex workers are still far from clear.

**Methods:**

Our study was based on data abstracted from a paper-and-pencil survey administered among 218 female street-based sex workers in Shanghai, China. We focused on self-reported client-initiated violence and intimate-partner violence in emotional, physical, and sexual forms. Social networks were characterized by the size and sources of financial and psychosocial support (e.g. family, friends, and peers). Multi-variable logistic regression was used to estimate adjusted odds ratios (AOR) of each type of violence exposure by social network structure after the adjustment of age, education, and years in Shanghai.

**Results:**

The street-based female sex workers in our study were primarily rural-to-urban migrants (95.7%) with an average age of 41 years old. 24.3% and 62.8% of the sex workers reported intimate-partner violence and client-initiated violence respectively. Lack of financial support, as defined by having only one individual or none in her peer support system to help financially, was significantly associated with self-reported intimate-partner violence (AOR: 2.5; 95% CI: 1.1–5.9). Respondents who reported client-initiated violence, by contrast, were more likely to report lacked psychosocial support from family (AOR: 2.2, 95% CI: 1.0–4.6) and peers (AOR: 5.1, 95% CI: 2.2–11).

**Conclusion:**

This study is one of the first to systematically analyze the associations between social network and gender-based violence among street-based female sex worker. We reported a high prevalence of both types of gender-based violence and their complex associations with family, friends, and peer support network. Policies with goals to reduce violence against women may apply these findings to leverage social network in the interventions against gender-based violence.

## Introduction

A considerable body of research has focused on the violence experienced by female sex workers globally [1–6]. A meta-analysis of this literature found that, on average, between half and two-thirds of sex workers had experienced gender-based violence in their life, most often intimate partner violence (IPV) or client-initiated violence (CIV) [2]. Yet, while research continues to find high victimization rates among female sex workers, this has not translated into better strategies or policies for reducing violence against them. Few national or international anti-violence policies or interventions actually include, discuss, or seek to mitigate the risks faced by female sex workers [2,7–9]. As a result, female sex workers have limited social and legal resources for addressing the health consequences of their victimization [10,11].

And, these consequences of gender-based violence can be numerous. Gender-based violence has been linked to immediate and long-term physical injury, unwanted pregnancy, abortion, gynecological complications, posttraumatic stress disorder and depression, and chronic pain among victims (often by examining IPV) [12–16]. Victims may turn to alcohol and drug use to cope with such experiences [17,18]. The health impacts of such violence is further complicated among sex workers given their unique occupational hazards and activities. Repeated and frequent sexual intercourse can increase the risk of internal vaginal and rectal tearing, increasing the risk of sexual transmitted infections, including HIV/AIDS and HCV [19–22]. Additionally, female sex workers with a history of psychosocial distress were less likely to require clients to use condoms, and engaged in more risky sex practices overall, further elevating their risk of exposure to sexual transmitted diseases including HIV/AIDS [17,18,23–25].

Female sex workers’ violence exposure is further compounded by the criminalization and stigma associated with their work. On average, between half and three-fourths of female sex workers report experiencing client-initiated violence, including physical and sexual assault, robbery, and kidnapping [2,5–7,19,26,27]. In their cohort study, Potterat and others found that sex workers in the United States were 18 more times likely to be murdered while on the job than similarly aged women of the same race who were not involved in sex work [27]. Such occupational experiences with violence are unlikely to be reported to police, especially in areas where prostitution is criminalized [7,21,28], and, indeed, sometimes such acts are actually perpetrated by police [3,21,28–30].

Many female sex workers contend with IPV but also client-initiated violence on a regular basis. These two types of violence are seldom considered independently or comparatively [2]. Instead, Deering and colleagues (2014) found that most research on female sex workers’ victimization focused either exclusively on CIV or did not distinguish between IPV and CIV, grouping them together on the basis of intimate contact with sex workers. However, as other researchers have noted, “intimate partner” is more involved than sexual contact, and evokes shared life commitments, goals, and responsibilities[31]. Clients, who may only interact with a female sex worker once, do not appear to meet this more nuanced definition. CIV, then, may be more similar to acquaintance or stranger violence rather than IPV. As Deering and colleagues note, such failure to separate these two types of violence may obscure important correlates and ignores theoretical distinctions that can inform interventions.

Among those theoretical distinctions is the protective role of social support in reducing victimization. Links between social support and improving health-related quality of life during stressful situations has been consistently found [32–36]. Quantitative research has particularly identified that social support reduces the long-term harm of IPV and can be crucial in enabling victims to leave their abusers [22,37–44]. Basic components of social support include attachment, emotional support, self-esteem building, provision of information and tangible assistance [35,44–47].

Previous research has established that when considering the impact of social support on reducing IPV is largely dependent upon the victim’s socio-cultural identity [22,37,44]. Groups that face considerable social stigmatization, including LGBT people, ethnical minorities, and migrants, are more likely to draw upon within-community support when facing stressful situations, including leaving violent relationships [22,37,44,48–55]. In some cases, such social identities are valued even over familial bonds, suggesting a dependence upon others with shared experiences of marginalization. Yet this same theoretical approach to mitigating and mediating IPV has not been tested among other socially stigmatized groups, including sex workers. Quantitative research suggests the lower levels of perceived social support by victims of IPV translate into higher levels of victimizations, suggesting that the size of a victim’s social support network may also be crucial[56–58]. Hobfoll & Lilly (1993) further hypothesized that tangible social support, such as financial and housing support, were essential for socially disadvantaged victims. Without the availability of tangible support within their social support network, Hobfoll and Lilly suggested that experiences of stress were exacerbated and women were less likely to leave violent situations[59].

These same social support theories have received only passing application to CIV. Recent qualitative research has indicated that female sex workers in the United States, United Kingdom, India, Mongolia, and China rely upon social support from fellow sex workers to reduce their exposure to CIV, indicating perhaps a similar pattern of seeking aid from individuals with shared socio-cultural background In the present study, we examined the impact of emotional and financial support and the size of the network on both IPV and CIV among a sample of Chinese female street-based sex workers (SBSW). We focus on Chinese SBSW since much of the research on violence against sex workers has been carried out in developed Western countries such as the United States, England, and Canada [3,6,60,61]. Additionally, the recent expansion of commercial sex markets in developing countries, such as Thailand, India, and China, has incentivized researchers to shift their study settings [40,41,62–67]. China, in particular, has experienced a considerable growth in the prostitution sector over the past three decades [40,41,67]. Hong and colleagues (2014) recently examined rates of both CIV and IPV among Chinese female sex workers and found high rates of both—nearly sixty percent had experienced IPV and forty-five percent reported CIV. However, as the authors note, to facilitate better response rates, they largely concentrated recruitment on indoor, brothel-based sex workers, oversampling female sex workers who worked in higher-income commercial sex venues. Since female street-based sex workers (SBSW) are usually poorer, less educated, and lack of protections of gate-keepers when compared to indoor sex workers, they often have the highest rates of victimization [63,64,68–70].

We hypothesize that having a larger social support network will generally lead to less victimization among SBSW overall. Specifically, in line with past research, we anticipate that social stigmatization will prompt SBSW to rely more upon their peer network, making peers important in mitigating both CIV and IPV. And, as a resource-uncertain population, we further hypothesize that more financial support will be correlated with less victimization, of both types. We conclude by discussing the implications of our results for interventions.

## Methods

### Study Site

Shanghai is China’s second largest city with 18 million residents and 4 million rural-to-urban migrants as of 2010. This confluence of wealth and travelers makes Shanghai an ideal location for sex work; as many as 200,000 FSW operate out of various venues throughout the city [69]. As in other major Chinese cities, many of Shanghai’s SBSWs are migrants, moving from China’s inland rural areas in the North and West to its industrial and financial centers in the South and East coast areas in search of better work opportunities [40,64,67]. For this survey, all participants were recruited from the Zhabei District which hosts the Shanghai railway station, the main point of entry for migrants in the city.

### Study Population

For many women, this rural-to-urban migration was an opportunity to leave poverty and abusive relationships behind [71]. Yet, upon arriving to Shanghai, many women found a saturated cheap-labor market. Prostitution, then, provided an immediate financial return when confronted by the lack of other job opportunities in the city. Most would not tell their family the nature of their work, even while continuing to send money back to their hometown for child and elder care. Compounded with the lack of *hukou*, local resident status, which excluded them from access to education, welfare, and medical insurance, these women reported a perilous existence, often surviving without financial and social safety nets. Unsurprisingly then, during qualitative interviews, women reported small social support networks, truncated by both distance from their home community and intersecting experiences of stigma.

Amplifying this isolation was their age. SBSW in Shanghai often tended to be older. In our qualitative study, most of the women we encountered were 35 and over. Few research studies have addressed the experiences of older sex workers specifically [63]. Previous research suggests that older Chinese FSWs had lower rates of education, worked in less economically desirable venues, faced more HIV risks, and experienced more regular partner victimization than middle-aged FSWs [61,63,68,72]. More generally, older women who experienced IPV were less likely to disclose their victimization to family members, instead relying upon friends as an extended support network [58]. Additionally, the interaction between age and migration status may further expose older SBSW to victimization. In their recent Canadian study involving SBSW, Goldenburg and colleagues (2014) found that occupation-related mobility and migration disrupted FSWs’ ability to establish community-based relationships, both with individuals and service organizations. Thus, older SBSW who are migrants are likely to face high rates of victimization with few local support systems of any type [73].

### Sampling

Our analysis draws upon a broader dataset examining the lives and working conditions of older street-based FSWs in Shanghai, conducted between 2011 and2012. Respondent driven sampling (RDS) was used to recruit participants. RDS is a modified form of chain referral sampling method that recruits individuals through their social networks [74–76]. Recent reviews have shown that RDS is an effective technique to sample most-at-risk populations for biological and behavioral surveys [71,77].

Researchers, with the help of staff members of Shanghai PiaoXue, a non-government organization (NGO) that serves high HIV-risk populations, identified seeds during the qualitative data collection phase before the survey study. Eight SBSW were recruited to serve as “seeds” for this study (4 migrants and 4 non-migrants). Eligible participants (including seeds) needed to be: (1) biologically female; (2) 18 to 65 years of age; (3) able to provide verbal or written consent in Mandarin; (4) self-identified as a current commercial sex worker (having sex with men for money or goods); and (5) primarily street-based in their solicitation of clients. As such, participants were not regularly working at any indoor venue (e.g., salon, karaoke bar, or massage parlor).

Following a standard RDS process, each participant who served as a seed was asked to refer three other SBSW to participate in the study [76]. Surveys and interviews were conducted at either a public cafe or at the office of Shanghai PiaoXue, at the participant’s discretion. Each recruit who finished the questionnaire was also given three coupons to distribute. Each coupon had a unique code linking the recruit to her recruiter. When the referee finished the survey, the recruiter received a $5USD reimbursement incentive.

### Ethic Statement

Recruited participants were verbally informed of the nature and purpose of the study, interview procedures, sensitive nature of the questions, confidentiality parameters, reimbursement for travel and time spent in the study, voluntary HIV/STI testing, risks and benefits (including referrals to other needed services), and the freedom to cease participation at any time without penalty. When respondents have verbally indicated an understanding of these issues, they would then sign a consent form, a copy of which will be given to the respondent and two copies of which will be placed in the project files. All interview instruments and study protocols were approved by the Institutional Review Boards of both Georgetown University and Fudan University. Each participant received $15 USD in cash as compensation for their time and travel expenses, as well as a pre-packaged health education resource kit.

### Measures

Client-initiated violence was defined as verbal, emotional, or physical violence inflicted by a client in the past six (6) months. Participating SBSW were asked to identify whether clients had ever: a) thrown something at or hit her; (b) withheld money (such as payment from her); (c) forced her to have sex with someone against her will; or (d) verbally insulted or yelled at her. Based upon these responses, a dichotomous variable was created to determine whether a respondent had experienced CIV.

Comparatively, intimate partner violence was defined as violence inflicted by the respondent’s current romantic or regular non-paying sexual partner (either husband or boyfriend). Eight questions were asked to SBSW whether their partners had: (a) thrown something at or hit her; (b) withheld money (such as income) from her; (c) forced her to have sex with someone against her will; (d) threatened to no longer help in term terms of finances or housing; (e) threatened to hurt her family or friends; (f) intentionally destroyed personal property (e.g.: cell phone); or (g) threatened to tell others that she was a sex worker. A dichotomous variable was created based upon these responses to identify whether the FSW had experienced any of these forms of IPV.

Social support was defined as “assistance and protections given to individuals” and can include components of emotional and tangible (financial) aid [44,78]. We measured social support using the Social Support Rating Scale (SSRS)[79]. Cultural adaptation of SSRS has been undertaken in China and has been applied in a wide range of Chinese populations because of its high reliability and validity (alpha = 0.92)[80–84]. To access financial support and network size, women were asked “If you encounter emergency situations, where do you obtain economic support? Multiple choices included spouse or boyfriends, other family member, friends, relatives, or peers. To measure emotional support and network types, participants were asked, “If you encounter a crisis, what are your resources for comfort and care?” with the same options in answers. Number of support sources refers to the total number of sources in SBSWs’ support network selecting from the options of a) zero sources; b) one source; c) two sources; or d) three or more sources.

Other socio-demographic variables include age, education level, *hukou* (resident status), years in Shanghai, marital status, average monthly income, and fluency in the Shanghai dialect. The final regression controlled for the effects of age, educational attainment, and years in Shanghai.

### Statistical Analysis

All statistical analysis was conducted using SAS 9.3 (Cary, NC). Chi-square and fisher exact tests were used to examine relationships between all categorical variables. Continuous variables, specifically age and years in Shanghai, were analyzed using analysis of variance (ANOVA). Crude and adjusted models were then used to study the relationship between venue characteristics and social support with client-initiated violence and IPV. Associations that were statistically significant (p<0.05) in the bi-variate analysis were used included as independent variables in the multivariable logistics regression model after checking the existence of co-linearity. Adjusted odds ratios and 95% confidence intervals of social support to predict violence outcomes were calculated after the adjustment of age, education, income, and language.

## Results

Forty-four participants of 262 SBSW did not complete the victimization checklist. This left us with a remaining sample size of 218. Non-respondents did not differ from the study sample in education, income, years in Shanghai, or based on other socio-demographic characteristics.

### Demographics

About a quarter (24.3%) of the sample reported experiencing IPV in their current relationships (Table 1). Comparably, 62.8% of SBSW reported experienced CIV in the past six months. There was little overlap between these experiences; just 16.5% of respondents had recently experienced both CIV and IPV (Table A and Table B in S1 File). Demographically, SBSW who reported violence experience of either type did not significantly differ from those who had not (Table 1). Collectively, reports of CIV and IPV were not significantly associated with one another (p-value: 0.66) among SBSW in our study.

**Table 1:** Socio-demographic characteristics of street-based female sex workers experiencing intimate partner violence and client-initiated violence in Shanghai, China 2011–2012 (N = 218).

	Total SBSWs (n = 218)	Reported Intimate-partner Violence (n = 53)	Reported Client-initiated Violence (n = 137)
**Victimization Rates,** %(N)	——	24.3% (53)	62.8% (137)
**Age,** Mean (SD)	41.0 (6.7)	43.6 (6.1)	40.6 (7.0)
**Migrant Status,** %(N)			
Migrant	95.7% (198)	25.8% (51)	63.6% (126)
Non-Migrant	4.3%(9)	0 (0)	66.7% (6)
**Marital Status,** %(N)			
Never Married	2.8% (6)	1.9% (1)	1.5% (2)
Married	61.2% (131)	67.3% (35)	62.2% (84)
Divorced/Widowed	35.9% (77)	30.7% (16)	36.3% (49)
**Education,** %(N)			
Elementary School or lower	36.8% (75)	21.3% (61)	62.7% (47)
Middle School or higher	63.2% (129)	27.1% (35)	59.7% (77)
**Monthly Income,** %(N)			
Less than 1000Y	4.1% (9)	33.3% (3)	33.3% (3)
Y1000.00-Y2999.99	40.1% (87)	31.0% (27)	62.1% (54)
Y3000.00-Y4999.99	41.5% (90)	18.9% (17)	63.3% (57)
> Y5000	14.3% (9)	19.3% (6)	71.0% (22)
**Years in Shanghai, Mean (SD)**	4.91 (3.4)	5.6 (3.4)	4.7 (3.2)
**Engagement in Sex Work,** %(N)			
Full-time	37.3% (78)	28.2% (22)	69.2% (54)
Part-time	62.7% (131)	20.6% (27)	60.3% (79)

All p-values were greater than 0.05 using chi-square, ANOVA (means), and Fisher Exact test when comparing socio-demographic characteristics between those who reported IPV vs. no IPV, and CIV vs. no CIV.

As a group, the SBSW in our study has a mean age of 41.0 (SD: 6.7) years old (Table 1). Most did not have a Shanghai *hukou* (residency) (95.7%). More than half (61.2%) of the study participants responded that they were married. Over thirty-five percent were divorced or widowed. Less than three percent (2.8%) had never been married. Some of the married respondents were living apart from their husbands due to migration to Shanghai. More than one-third of the SBSW (36.8%) had an elementary school education or lower.

SBSWs reported a large income range. Most indicated that they made between Y3,000-Y4,999 per month (41.5%) in 2012, above the average monthly income among Shanghai residents (Y2,431)[85]. Another forty percent made slightly less than that, between Y1000-2999 monthly, and 14.3% made Y5000 or more a month. Four percent made less than Y1000 per month. However, for most women, sex work was not their only source of income; most (62.7%) indicated they had other income sources besides prostitution. Full-time sex workers were more likely to be in the highest income bracket, making over Y5000 a month.

We found no significant differences of demographic characteristics between those who reported any violence experience and their counterparts. However, when we further categorized violence as no violence, IPV-only, CIV-only, and both, respondents who reported experiencing both types of violence were on average five years older than those who reported no violence (45±8 yrs.vs.40±7 yrs., p = 0.03;Table B in S1 File).

### Victimization

Among SBSW who reported CIV, the most commonly reported experiences were verbal abuse (62.8%), followed by withholding money (42.3%), and physical abuse (38.0%) such as a client hitting or throwing objects at them (Table 2). Slightly over five percent indicated that a client had forced them to have sex in the past six months.

**Table 2:** Types of violence reported by female street-based sex workers who experienced intimate-partner violence or client-initiated violence in Shanghai, China from 2011-2012(N = 218).

% (N)	Intimate-partner Violence	Client-initiated Violence
Hitting and throwing object at me	75.5 (40)	38.0 (52)
Withheld money (as payment or support) from me	28.3 (15)	42.3 (58)
Verbally insulting or yelling at me	N/A	62.8 (86)
Forced me to have sex with someone against my will	1.1(2)	5.1 (7)
Threaten to no longer help you in terms of finances or housing	73.6 (39)	N/A
Threaten to hurt my family or friends	41.5 (22)	N/A
Intentionally destroys my personal property (e.g.: cell phone)	56.6 (30)	N/A
Threatens to tell other that I am a sex worker	13.5 (7)	N/A

*Categories marked “N/A” were not measured in the questionnaire relating to the specific type of violence.

The most commonly reported forms of IPV included physical abuse (75.5%), partners threatening to withhold housing or financial assistance from respondents (73.6%), damaging property (56.6%), threatening to harm respondents’ loved ones (41.5%), stealing money (28.3%), and threatening respondents to public expose their profession (13.5%). Sexually assault was very rarely reported in relation to IPV (1.1%; Table 2). IPV-related violence also appears to include more attempts to psychologically control the respondent, most often by threatening their friends, family, or reputation.

### Impact of Financial & Psychosocial Support

The number of people in a SBSW’s financial and social support network had little impact on her likelihood to report IPV or CIV. However, those who have one or fewer sources of financial support were 2.5 times more likely to report IPV compared to those with a larger network of financial support (AOR = 2.5, 95% CI 1.1–5.9; Table 3).

**Table 3:** Multi-variable regression of client-based and intimate-partner violence by social support network size among female street-based sex workers in Shanghai, China from 2011–2012 (N = 218).

	Intimate partner Violence			Client-initiated Violence		
	%(N)	OR(95% CI)	AOR (95% CI)*	%(N)	OR(95% CI)	AOR (95% CI)*
**Financial Support**						
<1 Source	60.4 (32)	**3.0 (1.4–6.5)**	**2.5 (1.1–5.9)**	52.6 (40)	0.5 (0.2–1.0)	0.6 (0.3–1.4)
2 Sources	17.0 (9)	0.5 (0.2–1.3)	0.6 (0.2–1.7)	68.7 (55)	1.0 (0.5–2.0)	1.0 (0.4–2.1)
3+ Sources	22.7 (12)	Reference	Reference	68.9 (42)	Reference	Reference
**Psychosocial Support**						
< 1 Source	39.3 (22)	1.4 (0.5–2.6)	1.0 (0.4–2.4)	60.7 (34)	0.9 (0.4–2.0)	1.3 (0.5–3.0)
2 Sources	9.6 (10)	0.2 (0.1–0.5)	0.2 (0.7–0.5)	64.4 (67)	1.0 (0.5–2.0)	1.1 (0.5–2.3)
3+ Sources	35.1 (20)	Reference	Reference	61.4 (35)	Reference	Reference

*Adjusted by years in Shanghai, education, and age

In comparison, sources of financial or psychosocial support had a more notable impact on reporting violence. Having financial and social support from peers was the single largest protective factor against IPV (AOR for lack of financial support from peers = 2.6, 95% CI; AOR for lack of social support from peers = 5.1, 95% CI = 2.2–11.8; Table 4), but it made no significant difference on a participant’s experience with CIV. Conversely, a lack of family psychosocial support made FSWs 2.2 times more likely to report CIV (95% CI = 1.1–4.3; Table 4).

**Table 4:** Multi-variable regression of intimate-partner violence and client-initiated violence by sources of social support among female street-based sex workers in Shanghai, China, 2011–2012 (N = 218).

	Intimate Partner Violence		Client-initiated Violence	
	OR (95% CI)	AOR (95% CI) *	OR (95% CI)	AOR (95% CI) *
**Lack of Financial Support from**				
Boyfriend/Spouse	0.5 (0.2–1.1)	0.4 (0.2–1.1)	1.5 (0.7–3.4)	1.1 (0.7–4.0)
Relative	1.5 (0.8–3.1)	1.7 (0.8–3.7)	0.4 (0.2–0.8)	0.4 (0.2–0.7)
Family	0.3 (0.1–0.6)	0.3 (0.1–0.6)	1.7 (0.9–3.4)	1.6 (0.8–3.4)
Sisters/peer SBSW	**3.2 (1.6–6.6)**	**2.6 (1.2–5.7)**	0.7 (0.4–1.3)	1.0 (0.5–2.0)
Friends	1.9 (0.9–3.9)	1.8 (0.8–3.8)	0.8 (0.4–1.5)	1.1 (0.6–2.1)
**Lack of Psychosocial Support from**				
Boyfriend/Spouse	0.6 (0.3–1.4)	0.6 (0.2–1.4)	2.3 (1.1–4.8)	2.5 (1.1–5.6)
Relative	0.7 (0.4–1.4)	0.9 (0.4–2.0)	1.1 (0.6–2.0)	1.0 (0.5–1.9)
Family	0.2 (0.1–0.4)	0.2 (0.07–0.4)	**2.2 (1.1–4.3)**	**2.2 (1.0–4.6)**
Sisters/peer SBSW	**6.0 (2.8–12.7)**	**5.1 (2.2–11.8)**	0.4 (0.2–0.8)	0.5 (0.1–1.0)
Friends	0.6 (0.3–1.3)	0.5 (0.2–1.0)	0.7 (0.4–1.3)	0.9 (0.4–1.6)

*Adjusted for years in Shanghai, education, age, and income

### Limitations

There are several limitations that impact the generalizability of our findings. Response bias may play a role in this study[86]. Indeed past research with Asian participants has indicated high response bias and general aversion to discussing both domestic violence and sexual victimization with both researchers and social service agencies [87,88]. As such, subjects may have unconsciously underreported their experiences with violence and victimization due to their cultural background. IPV among married couples is extremely high in rural China and often considered a social norm [89,90].

Similarly, our sample reported a low prevalence of sexual victimization. We believe this reported low rate may be twofold. First, our question asked participants to report victimization within the last six month, again, rather than lifetime. Second, we did not use a checklist approach, instead asking if respondents had experienced force sex. As such, we expect that both our estimates of IPV and sexual assault are conservative. Future research should expand upon our approach by incorporating a stepwise or checklist form of questioning, along with adding a lifetime question, to gather more accurate rates.

Our study also relied upon a truncated RDS-frame, resulting in a relatively small sample (n = 218), rendering our findings not generalizable to the broader street-based sex worker population. Related to this, our sample is older than other studies because of the initial project’s intent to focus on middle-aged or older SBSW. However, we ultimately believe this oversampling of older and middle-aged SBSWs is a benefit, given their relative underrepresentation in most sex work research. Still, our results should not be interpreted as generalizable. We encourage others to replicate this research with samples of younger SBSWs and SBSWs in other geographic contexts. Finally, since the study was cross-sectional, we were only given an estimate of association between social support and violence and not the causation pathway. We recommend a large longitudinal study to further explore the relationships identified here.

## Discussion

This study expands upon recent research, notably by Hong and colleagues, to broaden public health conversations about violence faced by SBSW. Our study population is comprised of some of the most vulnerable and marginalized people engaged in commercial sex work—older migrant women working outdoors. And as has been found with other comparison of indoor-outdoor sex worker populations, our sample experienced higher rates of CIV than the indoor sample surveyed by Hong and colleagues [40]. Unlike Hong’s sample, our respondents were currently experiencing far more CIV (62.8%) than IPV (24.3%). (By comparison, in Hong and colleagues’ study, the findings were virtually flipped with approximately 58% of indoor female sex workers reported IPV and just 24% reported CIV.) Our qualitative research involving this same sample indicates that many SBSW cited marital disputes or violence as a reason for migrating to Shanghai [67]. As a group, then, these women may have been more vigilant against engaging in a second abusive relationship or less likely to disclose. We do expect, however, that had our IPV question had addressed lifetime, rather than just current experiences, that more of our study participants would have indicated victimization, similar to Hong’s study.

While the Hong study examined the impact of victimization on psychosocial distress, we looked at the relationship between financial and psychosocial support on victimization. As hypothesized, a SBSW’s social network did play a role in reducing her likelihood of victimization. As with other stigmatized communities, peer-support emerged as the single largest protective factor against IPV, but had no impact on CIV. Meanwhile, familial support had no impact on IPV, but did reduce CIV. Surprisingly, the size of a respondent’s support network did not necessarily reduce victimization although having a very small network—one or fewer individuals—was a risk factor for experiencing IPV.

Previous research has suggested that intimate bonds, including those with peers and family members, are protective against IPV generally [38,42,91]. Other research has indicated that peer support can mitigate client-initiated violence though our own findings did not reflect this [65,92]. Instead, among our sample of Shanghai-based SBSWs, peer support—both financial and psychosocial—emerged as the most crucial factor in reducing her risk of IPV. For the women in our sample—rural-to-urban migrants who are separated from their families—local peers may be the most physically accessible network that can provide both types of support.

The importance of peer support may be further amplified by the stigma and criminalization faced by female street-based sex workers. Our sample includes women who are among the most marginalized in Chinese society; they face more risk of arrests and generally lack the protections afforded indoor sex workers [63]. As migrants, they may be further marginalized in Shanghai, and not have the knowledge or ability to access structural protective systems, such as domestic violence programming. Criminalization and police-initiated violence against sex workers further isolates FSW and reduces their likelihood of reporting IPV to law enforcement. In this environment, then, peers become the most accessible support network, not just socially, but geographically.

Given how criminalization and social stigma negatively impact sex workers, best practices for reducing IPV among SBSW should incorporate population-specific findings. Though other research has often highlighted that other relationships—primarily family and friends—can reduce IPV victimization, our findings suggest that bolstering such relationships may not be protective for street-based sex workers, particular those who are migrants Instead, anti-domestic violence programming designed for sex workers must include and involve peers. Other research on collective peer empowerment among female sex workers in south India also noted how social stigma and criminalization fostered self-sufficiency among sex worker communities [93].

While family support did not have a significant impact on reducing IPV, a lack of familial psychosocial support did lead to a higher likelihood of experiencing CIV. A majority of our sample reported CIV (62.8%), while only a quarter (24.3%) had experienced IPV. Compared to Chinese women generally, our findings suggest that street-based FSW experience similar rates of IPV [89]. SBSW, then, appear to be much more likely to experience violence as a consequence of their occupation, rather than within their personal intimate relationships.

The role of family psychosocial support in reducing this exposure, then, is especially noteworthy. Family support, particularly in Chinese culture, is crucial in building a sense of self-worth. SBSWs with more family support, then, may experience a greater sense of self-worth and be less likely to engage with potentially violent (or otherwise risky) clients, thus ultimately experiencing less CIV. A parallel qualitative study suggests stigma surrounding drug use and commercial sex work may lead to self-isolation among SBSW, and the gradual reduction of their social support networks [67]. Having more family support, then, may be tied to SBSW experiencing less stigma or shame, and instead encouraging a sense of self-worth.

The connection between self-worth, stigma, and risky sexual behavior has not been well-documented among FSW, but in past studies involving men who have sex with men (MSM) self-esteem was linked to a strong social support [94]. And, similar studies with MSM have found that having higher self-esteem can reduce sexual risk-taking behaviors [94–99]. While few studies have examined how social stigma may impact SBSW’s engagement with individual clients, there is previous research indicating that broader communal stigma can reduce the likelihood that a female sex worker will seek out social or medical support [100].

Conversely, this same pathway could encourage more reporting among SBSW who enjoy more familial support[86]. More research on the connection between self-esteem and risk behaviors among SBSW is warranted. Overall, though, the lack of factors that impact CIV was surprising. This lack of finding, combined with the high prevalence, suggests that CIV, then, may not be tied to individual-level factors but structural ones [2,40,101]. The potential role of social stigma in exposing SBSW to CIV will not be remedied through self-esteem or financial management programming, particularly if such programming comes without an actual expansion of social support networks.

## Conclusion

Overall, a complicated picture of support for street-based FSWs emerges in this study. Specific sources and number of members in a SBSW’s social network play a complicated and imperative role in the prevention of IPV and CIV. Practitioners must consider the complexity of the quantity and sources in their targeted interventions. However, our research also indicates that best practices that address individual-level factors alone cannot completely eliminate violence against sex workers. Instead, structural factors, such as social stigma and criminalization, must also be addressed to reduce victimization. Practitioners can tailor interventions for intimate partner violence and client violence using specific social network ties. Furthermore, social support tailored programs may bridge cultural barriers, which is seen as an effective means to prevent consequence of violence such as PTSD, STI and HIV [37,102]. Interventions that address social stigma may also serve to improve survey underreporting as it may establish trust between the subject and interviewer.

## Supporting Information

S1 File Additional data analysis results. Demographic characteristics of the study sample by a dichotomized reported violence status (Table A). Demographic characteristics of the study sample by no reported violence, IPV only, CIV only, both IPV and CIV reported categories (Table B).
(DOCX)
