# Supplementary material for: Intimate-Partner and Client-Initiated Violence among Female Street-Based Sex Workers in China: Does a Support Network Help?
Source: PLoS One. 2015 Sep 28;10(9):e0139161. doi: 10.1371/journal.pone.0139161 (PMC4586135; doi:10.1371/journal.pone.0139161)
Supplement: S1 File — Demographic characteristics of the study sample by a dichotomized reported violence status (Table A). Demographic characteristics of the study sample by no reported violence, IPV only, CIV only, both IPV and CIV reported categories (Table B). (DOCX) [file pone.0139161.s001.docx]

**S1. Supplement Data**

**S1_Table A. Demographic characteristics among female street-based sex workers in Shanghai, China 2011-2012 (N=262).**

|  | Full Sample  (N=262) | No Violence 32.2 (73) | Violence 67.8 (154) | | *P* value |  |
| --- | --- | --- | --- | --- | --- | --- |
| **Age, years, Mean(SD)** | 41.2 (6.99) | 40.4 (6.4) | 41.1 (7.0) | | 0.4 (ANOVA) |  |
| **Migrant Status, %(N)** |  |  |  | |  |  |
| Migrant | 66.8 (175) | 95.7 (67) | 94.0 (141) | | 0.6 |  |
| Non-Migrant | 2.7 (7) | 4.3 (3) | 6.0 (9) | |  |  |
| **Marital Status, %(N)** |  |  |  | |  |  |
| Never Married | 4.3 (11) | 5.6 (4) | 3.3 (5) | | 0.10 |  |
| Married | 51.2 (132) | 69.0 (49) | 54.6 (83) | |  |  |
| Divorced | 39.1 (101) | 22.5 (16) | 35.5 (54) | |  |  |
| Widowed | 5.4 (14) | 2.8 (2) | 6.6 (10) | |  |  |
| **Education, %(N)** |  |  |  | |  |  |
| Less than Middle School | 39.0 (96) | 37.7 (26) | 42.3 (61) | | 0.5 |  |
| Middle School or Higher | 61.0 (150) | 62.3 (43) | 57.6 (83) | |  |  |
| **Monthly Income** |  |  |  | |  |  |
| Less than 1000Y | 6.1 (16) | 5.5 (4) | 4.6 (7) | | 0.05 |  |
| Y1000.00-Y2999.99 | 38.3 (100) | 32.9 (24) | 39.9 (61) | |  |  |
| Y3000.00-Y4999.99 | 41.0 (107) | 52.0 (38) | 35.3 (54) | |  |  |
| > Y5000 | 14.6 (38) | 9.6 (7) | 20.3 (31) | |  |  |
| **Years in Shanghai, Mean (SD)** | 5.22 (5.0) | 4.7 (3.65) | 5.3 (3.5) | | 0.6(ANOVA) |  |
| **Sex Work Engagement, %(N)** |  |  |  |  | | |
| Full-time | 38.5 (96) | 31.9 (22) | 44.6 (66) | 0.07 | | |
| Part-time | 61.4 (153) | 68.1 (47) | 55.4 (82) |  | | |
|  |  |  |  |  | | |

**S1_Table B. Comparisons of demographic characteristics of respondents who experienced no violence, IPV-only, CIV-only, and both among female street-based sex workers in Shanghai, China 2011-2012 (N=262).**

|  |  | *VIOLENCE OUTCOME* | | | | | |
| --- | --- | --- | --- | --- | --- | --- | --- |
|  | Full Sample  (N=262) | No Violence 38.8% (73) | IPV 11.7% (22) | | Client Violence 33.0% (62) | Both  16.5% (31) | *P-*value |
| **Mean Age, years (SD)** | 41.2 (6.99) | 40.15 (6.81) | 44.18 (4.48) | | 41.13 (7.09) | 44.76 (7.98) | 0.03 (ANOVA) |
| **Migrant Status, %(N)** |  |  |  | |  |  |  |
| Migrant | 66.8 (175) | 95.7 (67) | 100.0 (20) | | 93.4 (57) | 100.0 (31) | 0.51 (Fisher) |
| Non-Migrant | 2.7 (7) | 38.5 (3) | 0.0 (0) | | 6.6 (4) | 0.0 (0) |  |
| **Marital Status, % (N)** |  |  |  | |  |  |  |
| Never Married | 0.3 (11) | 5.6 (4) | 0.0 (0) | | 1.6 (1) | 3.3 (1) | 0.77 (Fisher) |
| Married | 51.2 (132) | 69.0 (49) | 68.2(15) | | 70.5 (43) | 66.7 (20) |  |
| Divorced | 39.1 (101) | 22.5 (16) | 31.8 (7) | | 21.3 (13) | 30.0 (9) |  |
| Widowed | 5.4 (14) | 2.8 (2) | 0.0 (0) | | 6.6 (4) | 0.0 (0) |  |
| **Education, %(N)** |  |  |  | |  |  |  |
| Less than Middle School | 39.0 (96) | 37.7 (26) | 27.3 (6) | | 41.1 (23) | 34.5 (10) | 0.71 |
| Middle School or Higher | 61.0 (150) | 62.3 (43) | 72.7 (6) | | 59.0 (33) | 65.5 (19) |  |
| **Monthly Income, % (N)** |  |  |  | |  |  |  |
| Less than 1000Y | 6.1 (16) | 5.5 (4) | 4.5 (1) | | 1.6 (1) | 6.4 (2) | 0.11 |
| Y1000.00-Y2999.99 | 38.3 (100) | 32.9 (24) | 33.8 (13) | | 32.8 (20) | 45.2 (14) |  |
| Y3000.00-Y4999.99 | 41.0 (107) | 52.0 (38) | 27.3 (6) | | 41.0 (25) | 35.5 (11) |  |
| > Y5000 | 14.6 (38) | 9.6 (7) | 9.1 (2) | | 24.6 (15) | 12.9 (4) |  |
| **Years in Shanghai, Mean(SD)** | 5.22 (5.0) | 4.88 (3.55) | 5.48 (3.73) | | 5.02 (3.08) | 5.92 (4.11) |  |
| **Sex Work Engagement, %(N)** |  |  |  |  | |  |  |
| Full-time | 38.5 (96) | 31.9 (22) | 38.1 (8) | 49.2 (30) | | 50.0 (14) | 0.17 |
| Part-time | 61.4 (153) | 68.1 (47) | 61.9 (13) | 50.8 (31) | | 50.0 (14) |  |
|  |  |  |  |  | |  |  |
|  |  |  |  |  | |  |  |
